# A Bedside Method for Measuring Effects of a Sedative Drug on Cerebral Function in Newborn Infants

**DOI:** 10.3390/s23010444

**Published:** 2022-12-31

**Authors:** Sofie Nilsson, Anton Tokariev, Marjo Metsäranta, Elisabeth Norman, Sampsa Vanhatalo

**Affiliations:** 1 Pediatrics, Department of Clinical Sciences Lund, Lund University, Skane University Hospital, 22185 Lund, Sweden; 2 BABA Center, Departments of Pediatrics and Clinical Neurophysiology, Children’s Hospital, Helsinki University Hospital Helsinki, 00029 Helsinki, Finland; 3 Department of Pediatrics, Helsinki University Hospital, University of Helsinki, 00029 Helsinki, Finland; 4 Department of Physiology, University of Helsinki, 00014 Helsinki, Finland

**Keywords:** brain monitoring, sedative drug, dexmedetomidine, newborn, EEG analysis

## Abstract

Background: Data on the cerebral effects of analgesic and sedative drugs are needed for the development of safe and effective treatments during neonatal intensive care. Electroencephalography (EEG) is an objective, but interpreter-dependent method for monitoring cortical activity. Quantitative computerized analyses might reveal EEG changes otherwise not detectable. Methods: EEG registrations were retrospectively collected from 21 infants (mean 38.7 gestational weeks; range 27–42) who received dexmedetomidine during neonatal care. The registrations were transformed into computational features and analyzed visually, and with two computational measures quantifying relative and absolute changes in power (range EEG; rEEG) and cortico-cortical synchrony (activation synchrony index; ASI), respectively. Results: The visual assessment did not reveal any drug effects. In rEEG analyses, a negative correlation was found between the baseline and the referential frontal (rho = 0.612, *p* = 0.006) and parietal (rho = −0.489, *p* = 0.035) derivations. The change in ASI was negatively correlated to baseline values in the interhemispheric (rho = −0.753; *p* = 0.001) and frontal comparisons (rho = −0.496; *p* = 0.038). Conclusion: Cerebral effects of dexmedetomidine as determined by EEG in newborn infants are related to cortical activity prior to DEX administration, indicating that higher brain activity levels (higher rEEG) during baseline links to a more pronounced reduction by DEX. The computational measurements indicate drug effects on both overall cortical activity and cortico-cortical communication. These effects were not evident in visual analysis.

## 1. Introduction

Newborn infants, born preterm and/or seriously ill, may need weeks of care at a neonatal intensive care unit (NICU) during a period characterized by significant hemodynamic instability and a rapidly progressing brain development with high sensitivity to environmental and therapy-related adverse effects. Prior studies have shown that neonatal pain or stress may have a negative impact on neurodevelopment shown as structural abnormalities [1,2] or abnormal neurobehavioral outcomes [3,4]. Analgesic and sedative drugs, mostly opioids, are often used to reduce pain and or stress during neonatal intensive care. However, these drugs have well-known immediate cardiovascular and respiratory side-effects as well as negative long-term effects related to apoptosis and reduced brain growth [5,6,7]. In order to reduce the opioid doses, alpha2-agonists (clonidine and dexmedetomidine; DEX) have recently been introduced as an adjunctive treatment in neonatal care [8,9,10]. These medications are considered safe; however, their use in the NICU has been “experience-based” rather than “evidence-based” as recommended by the European Medicines Agency, EMA [11].

Analgesic and sedative drugs have cerebral effects, and evaluation of drug exposure thus requires objective cerebral measurements to be added to non-cerebral variables such as behavioral response and cardiovascular effects. This is particularly important in newborn infants, as assessment of neurobehavioral responses including level of consciousness, vigilance or pain may be challenging. Electroencephalography (EEG) and amplitude-integrated EEG (a compressed visual display of EEG signals, aEEG) have been applied as an objective assessment of cerebral drug effects. Prior studies have shown that morphine and midazolam may cause depression of cortical activity [7,12,13,14]. A recent work by Cortes-Ledesma et al. suggested an overall and inter-individually variable reduction in brain activity after DEX administration [15]. In addition, there are unpublished data from infants treated with DEX suggesting that overall EEG abnormalities are disproportionately accentuated compared to their clinical appearance (Vanhatalo, unpublished observations).

Cerebral monitoring of the aEEG trend for seizure detection and evaluation of cortical activity, e.g., the presence of sleep-wake cycling is routinely performed in the NICU [16,17,18,19]. While aEEG is effective for a general qualitative clinical bedside assessment, the aEEG interpretation is still a subjective assessment with a substantial inter-rater variation and consequently poor sensitivity to those effects that may accompany drug treatment or other therapeutic maneuvers [18,19].

Aiming at evidence-based and safe drug treatment, studies with objective quantitative methods and a reduced risk for interpreter-related differences are needed. Two such studies in the neonatal context have recently been presented. Failla et al. described that cortical synchrony is affected by morphine using the Activation Synchrony Index [20] and van’t Westende et al. reported in a systematic review that quantitative EEG measures were associated with long-term outcomes [21]. However, no quantified computational measures have been published on the effects of DEX on cerebral function in newborn infants.

In this study we aimed to develop an objective and quantitative method for measuring cerebral effects of DEX administration. In particular, we aimed to capture two conceptually different mechanisms of brain function; the overall level of cortical activity and the level of interhemispheric communication as measured with range-EEG (rEEG) and Activation Synchrony Index (ASI), respectively; both are key components of healthy brain function in the conventional EEG interpretation [22,23]. Our hypothesis was that rEEG and ASI may disclose subtle effects on cortical activity that are context-sensitive, i.e., relative to the ongoing cortical activity by the time of drug administration.

## 2. Materials and Methods

EEG data were retrospectively collected from the archives at the neonatal intensive care unit, Children’s Hospital, Helsinki University Central Hospital, Finland. The recordings had been conducted between April 2013 and September 2016 in a group of preterm and term infants (*n* = 21) with a mean of 38.7 gestational weeks (range 27–42) who had received DEX for clinical purposes while being continuously monitored with EEG as part of their clinical care.

The recordings were performed with a NicOne EEG system (https://neuro.natus.com) (accessed on 15 November 2022), collecting four EEG signals with common references at Fz (frontal midline) and Cz (central midline); two frontal (F3 and F4) and two parietal channels (P3 and P4). The EEG sampling was performed at 250 Hz and we inspected frequency bands from 0.2 to 35 Hz, as this is the conventional band range in clinical EEG. From the EEG recordings of varied lengths, we selected 2-hour epochs before and after DEX administration.

### 2.1. Computational Analyses

**Figure 1:**
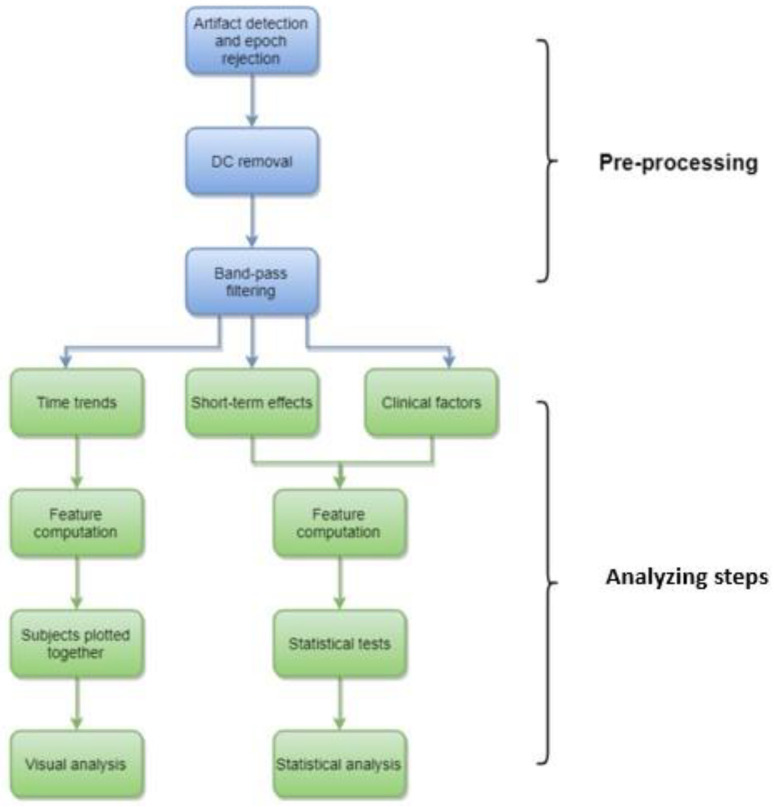
Study flowchart.

The full outline of the analytic process is shown in Figure 1. A thorough description of the methods and the detailed introductions to the chosen approaches have been thoroughly described before [24]. The full analytic scripts are also available from the authors at request. A brief description of the key aspects of the computational methods is as follows: The recordings were first reviewed visually for a rough data quality check (Figure 1). The signals in the recording montage were then exported into European Data Format and further analysis was performed using a custom-made Matlab script (https://www.mathworks.com) (accessed on 15 November 2022), which included preprocessing for automated artifact detection and removal. The artifact detection algorithm consisted of three separate functions: evaluating the amplitude as well as high and low frequency artifacts [24].

**Figure 2:**
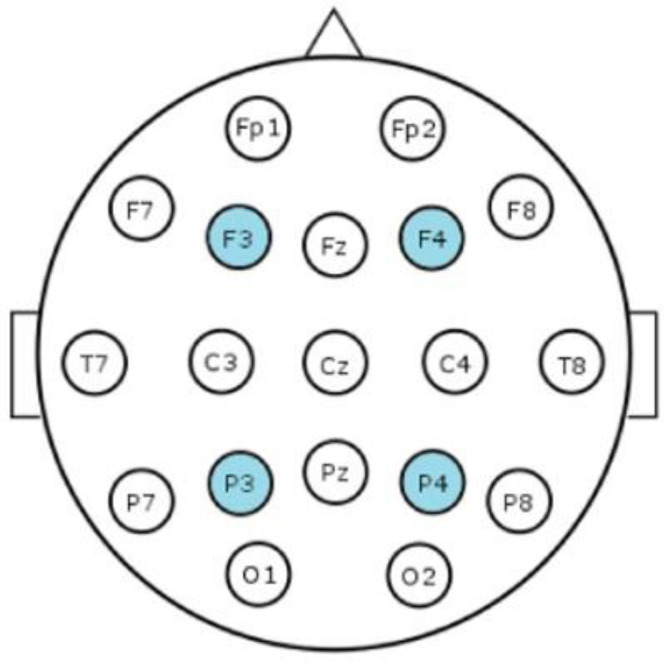
Positions of the electrodes. An illustration of the 10–20 system. The channels used in this study are highlighted in blue.

Two computational features were then chosen, both of which are computationally light and comparable with the currently used aEEG methods. First, the range-EEG (rEEG) [23] which is calculated as the minimum amplitude subtracted from the maximum amplitude for two second windows with no overlap, and for these analyses the running mean value was used. As compared to aEEG trends, the rEEG is emphasizing the amplitude variation (peak-to-peak) in the spontaneous EEG signals, which supports its utility in measuring amplitude levels [23]. Secondly, we computed the as yet only validated measure of interhemispheric synchrony, the Activation Synchrony Index (ASI) [22] which quantifies the co-occurrence of the activity bursts (called spontaneous activity transients SATs) between two EEG signals in both the preterm and the full-term infants [23,25,26]. The signal derivations used for this purpose were either referential (F3-ref, F4-ref, P3-ref, P4-ref) or bipolar (F3-P3, F4-P4, F3-F4, P3-P4) derivations in order to estimate intra- and interhemispheric synchrony, respectively. See Figure 2 showing the scalp locations of these electrodes.

The study consists of three different analyses:

- Long-term effects
- Short-term effects
- Clinical factors

#### 2.1.1. Long-Term Effects

The computational features were initially estimated for the entire four hours EEG-epoch to inspect possible long-term effects after the DEX administration. The individual recordings were divided into 2.5 min epochs and rEEG and ASI were computed for every epoch. The mean value of each epoch was then plotted into a time trend as a function over time for all the infants. These trends were then inspected visually to detect any clear prominent changes in the values.

#### 2.1.2. Short-Term Effects

**Figure 3:**
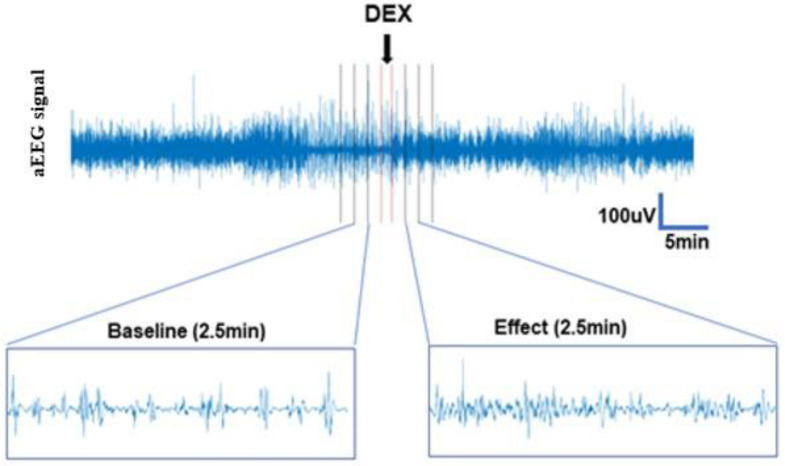
Short-term effects. Epochs of 2.5 min were created from the 12 and 10 min pre-(baseline) and post (effect) DEX administration periods. Due to uncertainty regarding the exact time of DEX administration, one-minute epochs before and after the administration were created, represented here as red lines.

Since it was not possible to verify the absolute timing of DEX administration in a retrospective dataset, we allowed 1 min gaps immediately before and after the time point which was noted as the time point for DEX administration. Due to a high number of artifacts during the baseline epoch this was extended from 10 min to 12 min, whereas the post-drug epoch remained 10 min. The individual recordings were divided into 2.5 min epochs and rEEG and ASI were computed for every epoch. The median of all epochs was calculated, and the pre-and post-drug administration values were compared (Figure 3).

We then investigated the amount of change in cortical activity during the post-drug period related to the baseline by subtracting the median baseline value from the median post drug change value (absolute change; delta value). For a detailed description of the methodology, see Suvisto [24].

#### 2.1.3. Clinical Factors

To rule out other factors that could affect brain activity, three different clinical factors were analyzed for interference: gestational age (GA), concomitant use of other drugs with central nervous effects (fentanyl), and the clinical diagnosis (Table 1).

To assess the correlation between the GA and the baseline for rEEG and ASI, the total number of days and median values, respectively, were used. The baseline median values of rEEG and ASI of the infants who did not receive fentanyl (*n* = 9) were compared to those of the infants who did receive fentanyl (*n* = 12). The last clinical factor to be tested was diagnosis, which was divided into four different categories based on the main diagnosis and these were compared to the baseline (Table 1).

### 2.2. Statistical Analyses

For statistical analyses, non-parametric tests were used; correlations were assessed with Spearman´s test (delta/baseline; GA/baseline), group comparisons were performed using Wilcoxon rank sum test (pre/post drug; fentanyl/baseline) and the multiple groups were compared using Kruskal-Wallis test (diagnosis/baseline). Multiple comparisons were a priori minimized to those tests that we considered to have a physiologically meaningful interpretation. We focused on a global level of brain activity in the rEEG assessment by investigating biparietal (P3-P4 derivation) signals and co-incident findings in both hemispheres (F3-F4, and P3-P4). The cortico-cortical activation synchrony was assessed only from the interhemispheric (F3-P3 vs. F4-P4) or intrahemispheric (F3 vs. P3 and F4 vs. P4) comparisons.

## 3. Results

### 3.1. Long-Term Effects

**Figure 4:**
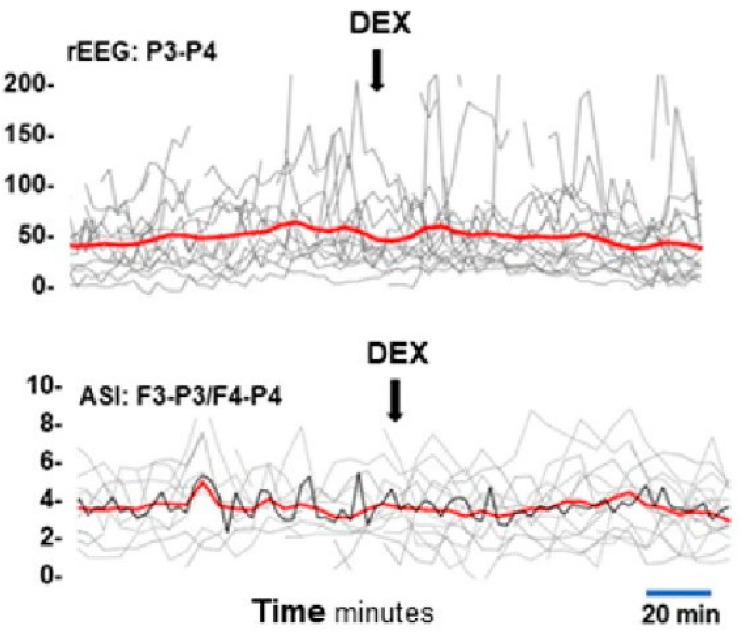
Time trend of rEEG and ASI. Time trend for rEEG from channel P3-P4, and ASI from channel F3-P3/F4-P4. Grey lines show the individual mean values, and the red line shows the mean values of the whole cohort during 2.5 min epochs.

Visual inspection of the aEEG trends of the 4 h recordings before and after DEX administration showed a random fluctuation, and neither rEEG nor ASI showed any consistent or longer standing changes (Figure 4).

### 3.2. Short-Term Effects

**Figure 5:**
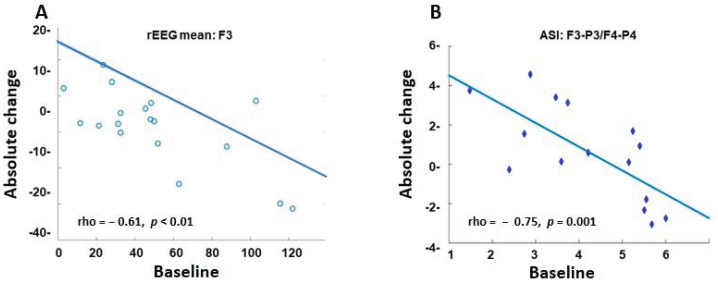
Correlation between the absolute change and the baseline. Scatterplot of the rEEG mean from channel F3 (**A**) and the ASI mean from channels F3-P3/F4-P4 (**B**) using Spearman´s test.

In the rEEG analyses, there were no statistically significant changes after DEX administration as compared to the baseline. However, the delta value compared to the baseline showed a significant negative correlation in referential frontal F3 (rho = −0.612; *p* = 0.006) and parietal P4 (rho = −0.440; *p* = 0.061) derivations. This indicates that higher brain activity level (higher rEEG) during baseline links to a more pronounced reduction by DEX (Figure 5A)

Concordant results were found in the ASI analyses where the delta value was strongly and negatively correlated to the baseline in the interhemispheric F3P3-F4P4 (rho = −0.753; *p* = 0.001) as well as the frontal comparison F3-F4 (rho = −0.496; *p* = 0.038) (Figure 5B).

### 3.3. Clinical Factors

The only significant result in the analyses of clinical factors compared to the baseline was the use of fentanyl, which was associated with a lower ASI (*p* = 0.0023).

## 4. Discussion

Our study results indicate that using computational measures for EEG-analysis can reveal otherwise non-detectable effects of sedative drugs, and in our case, the effects of DEX. The data shows that statistical analyses of computational measures demonstrate a negative correlation between the baseline and the effects of DEX administration which was not detectable upon visual examination of the time trends. Apparently, human assessment is not efficient enough to detect detailed differences in the time trends and robust quantitative measures are needed. We showed that treatment with DEX has effects on cortical activity in newborn infants, with drug-related effects being proportionate to brain activity prior to drug administration.

Out of nine different computational metrics presented in a master thesis by Suvisto et al. [24], we chose two of those considered most relevant for the neonatal population. First, based on prior literature showing a general reduction in brain activity following exposure to CNS-affecting drugs [7,12,13,14], we assumed that an amplitude-based index from the rEEG would capture the effect [23]. Secondly, as clinical experience of visual EEG reviews suggest a possible reduction of interhemispheric synchrony by DEX, we chose to employ the only validated measure of interhemispheric synchrony, the activation synchrony index (ASI) [22].

The initial visual trend analysis did not reveal any significant changes.

As expected in sedative care, our findings show that DEX administration influences newborn cortical activity. However, we could also show that the effect depended on the individual level of cortical activity at the time of drug administration. This effect was significant and could be quantified by using both an amplitude-based measure (rEEG) and the measure of cortico-cortical activation synchrony, ASI. It is by now widely established that cortico-cortical synchrony is a fundamental characteristic of brain function [26]. The burst level synchrony as measured more specifically by the ASI metric [22,25] is thought to reflect a developmentally essential communication mechanism in the early brain networks [26]. Our results indicate that synchrony in cortico-cortical networks (ASI) or the amount of brain activity in general (rEEG) can decrease only if there is a sufficient level of synchrony or activity amplitudes, depending on age and cerebral pathology and which must be considered when using these metrics clinically, or in research [26].

Our findings are in line with the only previous published study on the EEG effects of sedation with DEX in newborn infants; the findings by Cortes-Ledesma et al. who found prolonged interburst-intervals (IBI) and the disappearance of sleep-wake-cycling at administration of DEX [15]. This group also demonstrates reduced activity in regional saturation as monitored with Near Infrared Spectroscopy (NIRS) possibly related to local vasoconstriction [27].

This work should be considered a methodological pilot study, rather than a conclusive assessment of the clinical DEX safety. The weakness of this study is the lack of clinical data, which was obtained retrospectively from medical charts. The study group consisted of a small and very heterogeneous cohort of infants with different cerebral diagnoses, and 15 of the 21 infants received a combination of multiple drugs with unspecified doses, serum concentrations, and potentially different cortical effects. Only fentanyl was included in the analyses and the data on the correlations with clinical factors must be regarded insufficient.

Further clinical studies are needed, and more prospective studies are warranted. Such studies should be performed in an experimental setting; the computational EEG measures outlined in this study can provide the key translational bridge between findings in human infants to those obtained in animal models.

The computational measures presented in the present work can be fully automatized, and both the rEEG [23] and ASI [22] are shown to present well as long duration EEG trends; however, displaying the relative effects as shown in our present study will need more experimentation before clinical implementation. In an era with an increased focus on the importance of individualized care and drug dosing, monitoring of drug effects should be prioritized during neonatal intensive care. New algorithms based on computational measures of cortical activity, such as presented in this study, could be implemented in the clinical bedside monitors as additional trend curves to allow real-time cerebral surveillance of sedation and sedative drug effects. Such improvements would be valuable to optimize dose adjustments and thereby promote safer care of sick infants in the NICU.

## 5. Conclusions

The sedative effect of dexmedetomidine in newborn infants is shown as reduced cortical activity which escapes traditional visual EEG interpretation but can be quantified with computational measures. These analyses demonstrate that the higher the levels of brain activity and synchrony are before drug administration, the stronger reduction is linked to DEX administration. Further studies are needed to evaluate and develop computerized EEG analyses, and thereby promote reliable, objective monitoring of effective and safe drug use in the NICU.

## Figures and Tables

**Table 1:** Demograhics.

Clinical Characteristics	*n* = 21
Gestational age, weeks	38.7 (27–42)
Birthweight, g	3200 (1150–4780)
Postnatal age at study, day	2 (0–13)
Diagnosis groups; 0 = Other (MAS *, RDS *, PPHN *, infection) 1 = HIE * (mild or moderate) 2 = HIE * (severe) 3 = IVH *	5 8 6 2
Concurrent drugs affecting the brain during the examination (fentanyl, ketanest, phenobarbital, caffeine citrate)	15

* HIE (Hypoxic Ischemic Encephalopathy); IVH (Intraventricular Hemorrhage); MAS (Meconium Aspiration Syndrome); RDS (Respiratory Distress Syndrome); PPHN (Persistent Pulmonary Hypertension).

## Data Availability

The results are published in the MSc thesis of Samuel Suvisto http://urn.fi/URN:NBN:fi:aalto-201811135772 (accessed on 15 November 2022).

## Abbreviations

aEEG	Amplitude-integrated EEG
ASI	Activation Synchrony Index
DEX	Dexmedetomidine
EEG	Electroencephalography
EMA	European Medicines Agency
GA	Gestational Age
NICU	Neonatal Intensive Care Unit
NIRS	Near-infrared Spectroscopy
rEEG	Range EEG
